# Comparing the Keyto App and Device with Weight Watchers’ WW App for Weight Loss: Protocol for a Randomized Trial

**DOI:** 10.2196/19053

**Published:** 2020-08-17

**Authors:** Sean R Locke, Kaja Falkenhain, Dylan A Lowe, Terry Lee, Joel Singer, Ethan J Weiss, Jonathan P Little

**Affiliations:** 1 University of British Columbia Kelowna, BC Canada; 2 University of California San Francisco San Francisco, CA United States; 3 University of British Columbia Vancouver, BC Canada

**Keywords:** diet, low carbohydrate, mHealth, ketogenic diet, weight loss

## Abstract

**Background:**

Obesity and being overweight are major contributing factors for many diseases. Calorie restricted diets often fail to result in sustained long-term weight loss. Very low–carbohydrate, high-fat ketogenic diets have been suggested to have superior metabolic and weight loss effects. Keyto is a low-cost, highly scalable mobile health (mHealth) app paired with a noninvasive biofeedback tool aimed at facilitating weight loss through a personalized healthy and predominantly plant- and fish-based ketogenic diet.

**Objective:**

This protocol describes a randomized trial comparing the efficacy of the Keyto mHealth app and device intervention to that of Weight Watchers’ WW app in individuals who are overweight or obese. The primary outcome is weight loss after 12 weeks. Secondary and exploratory outcomes, including metabolic and cardiovascular risk factors, will be assessed at 12, 24, and 48 weeks.

**Methods:**

A total of 144 participants will be recruited and randomized to either the Keyto program or Weight Watchers program. Study participants will be guided through the study via video conference or phone calls and will undergo a fasting blood analysis performed by a third-party diagnostic lab at weeks 0 and 12 to assess metabolic and cardiovascular risk markers. All participants will be asked to weigh themselves daily on a study-provided Bluetooth-enabled scale. Participants randomized to the Keyto arm will also be asked to measure their breath acetone levels, a measure of ketosis, with the Keyto device 3 times per day.

**Results:**

Recruitment started in December 2019. Rolling recruitment is expected to be completed by July 2020. Data collection and analysis of the primary intervention phase is expected to be completed in October 2020. The 24- and 48-week follow-ups are expected to be completed in January 2021 and July 2021, respectively.

**Conclusions:**

This trial will provide high-quality evidence regarding the efficacy of the Keyto weight loss program in individuals who are overweight and obese in a free-living condition. This study also fills a gap by examining the impact of a ketogenic diet emphasizing plant- and fish-based fats on blood lipid profile and cardiovascular disease risk.

**Trial Registration:**

ClinicalTrials.gov NCT04165707; https://clinicaltrials.gov/ct2/show/NCT04165707.

**International Registered Report Identifier (IRRID):**

DERR1-10.2196/19053

## Introduction

Obesity is a major risk factor for a variety of diseases, including type 2 diabetes, cardiovascular diseases, musculoskeletal disorders, and some cancers [1]. It is estimated that over 40% of adults worldwide attempt weight loss diets each year [2]. Unfortunately, conventional calorie restriction diets often fail to produce long-term weight loss [3]. Therefore, novel dieting techniques must be explored in order to successfully treat obesity and lower disease risk.

Although many different dietary approaches can lead to weight loss [4], increasing evidence suggests that a very low–carbohydrate ketogenic diet may have superior metabolic and weight loss effects [5-10]. On a ketogenic diet, when carbohydrate intake is kept very low (<50 g/day), high rates of lipolysis increase delivery of free fatty acids to the liver, which converts them into ketone bodies (β-hydroxybutyrate, acetoacetate, and acetone) that the brain and body can use as an alternative fuel source. Accumulating evidence suggests that ketones mediate reduced feelings of hunger when individuals follow a ketogenic diet [11,12], which could help facilitate sustained weight loss. A ketogenic or low-carbohydrate diet may also be beneficial because it can potentially increase energy expenditure during weight loss maintenance [13]. When compared to other dietary approaches, the ketogenic diet also provides a unique opportunity to follow and track a biomarker in the form of ketone levels that may provide useful and actionable information.

The effectiveness of any diet is limited by an individual’s ability to adhere. Adherence to a diet requires considerable cognitive and self-regulatory resources daily at every meal or eating opportunity. Meta-analytic evidence suggests that self-regulatory behavior change strategies, such as self-monitoring, play a crucial role in increasing adherence to diet and healthy behaviors [14,15]. Very low-carbohydrate ketogenic diets can be particularly challenging to adhere to because of the complexity of understanding the carbohydrate content of consumed food and whether consuming certain foods will promote or limit ketosis [16,17]. However, the current approaches to self-monitoring ketogenic diets (eg, through finger stick blood monitors, urine ketone strips, or food logs) are typically expensive, painful, inconvenient, or inaccurate. There is emerging technology that can lower the burden of self-monitoring by measuring breath acetone to provide individuals embarking on a ketogenic diet with immediate ketone-specific self-monitoring that could help optimize adherence and success [18].

Keyto (Keyto Inc) is a scalable comprehensive weight loss program that combines resources (eg, recipes, searchable database, menus, meal plans, social support) delivered from a mobile health (mHealth) app with information from an accompanying breath acetone sensor to help individuals learn about and monitor whether they are in ketosis. Because a ketogenic diet is very low in carbohydrates but high in fat, there is some concern as to the impacts of such a predominantly high- (saturated) fat diet on cardiovascular health [19]. Keyto helps to guide users toward more heart-healthy plant- and fish-based fat sources to achieve ketosis. Thus, the goal of the Keyto program is to facilitate weight loss through a personalized healthy and ketogenic diet. As of January 2020, Keyto has delivered over 30,000 Keyto breath acetone analyzers to customers with reported anecdotal success, but robust evidence is required to evaluate the efficacy of this low-cost, scalable weight loss intervention. The purpose of this research is to conduct a pragmatic randomized trial to test the efficacy of the Keyto biofeedback and app intervention compared to Weight Watchers’ WW (Weight Watchers International Inc) diet app. The WW app was chosen as the active comparator as it is a widely accepted dietary approach with robust evidence for its effects on weight loss and cardiovascular health [20]. The hypotheses and exploratory research questions can be found in Table 1.

**Table 1:** Overview of study hypotheses and research questions.

Measures		Outcomes	Timeframe	Hypothesis or question
**Primary outcome**				
	Bodyweight scale	Change in body mass (in kilograms)	12 weeks	Those in the Keyto intervention arm will achieve greater weight loss at 12 weeks than those in the Weight Watchers arm.
**Secondary outcome**				
	Bodyweight scale			
		Change in body mass (in kilograms)	24 and 48 weeks	Those in the Keyto intervention arm will achieve greater weight loss at 24 and 48 weeks than those in the Weight Watchers arm.
		Change in body mass (in kilograms)	Daily weight measured during the first 12 weeks	What are the patterns of body mass change during the first 12 weeks between the 2 study groups?
	Automated self-administered 24-hour dietary recall			
		Carbohydrate intake in 24-hour period (in grams)	12, 24, and 48 weeks	Those in the Keyto intervention arm will report lower carbohydrate intake at 12, 24, and 48 weeks.
		Total fat, saturated fat, polyunsaturated fat, monounsaturated fat intake in 24-hour period (in grams)	12, 24, and 48 weeks	Does the Keyto intervention or WW app intervention influence fat intake from baseline to 12, 24, and 48 weeks?
		Total energy intake in 24-hour period (in kilocalories)	12, 24, and 48 weeks	We will examine changes from baseline to 12, 24, and 48 weeks in total energy intake in the Keyto and Weight Watchers groups.
	Venous blood sample	HbA_1_c^a^, fasting glucose, fasting insulin, fasting high-sensitivity C-reactive protein, fasting HOMA-IR^b^, fasting total cholesterol, fasting HDL^c^ cholesterol, fasting lipoprotein fractions, fasting lipoprotein (a), fasting triglycerides, fasting non-HDL cholesterol	12 and 48 weeks	We will examine changes from baseline to 12 and 48 weeks in metabolic and cardiovascular risk blood biomarkers in the Keyto and Weight Watchers groups.
**Exploratory outcome**				
	Venous blood sample	Fasting albumin, fasting globulin, fasting total bilirubin, fasting alkaline phosphatase, fasting aspartate aminotransferase, fasting alanine aminotransferase	12 and 48 weeks	We will explore changes from baseline to 12 and 48 weeks in blood biochemistry in the Keyto and Weight Watchers group.
**Manipulation checks**				
	Questionnaires			
		Food attitude survey	12, 24, and 48 weeks	We will examine all baseline questionnaires as potential covariates. We will also examine differences in questionnaire measures between the 2 groups at 12, 24, and 48 weeks.
		Cravings, mood, and energy	Weekly	
		Self-reported dietary adherence	Weekly	
		Pittsburgh Sleep Quality Index	12, 24, and 48 weeks	

^a^HbA_1_c: hemoglobin A_1_c.

^b^HOMA-IR: homeostasis model assessment of insulin resistance.

^c^HDL: high-density lipoprotein.

## Methods

### Overview of Protocol

This study is a 2-arm pragmatic randomized clinical trial. Participants will be randomized to one of 2 weight loss conditions: Keyto or Weight Watchers. The primary endpoint will be weight loss at 12 weeks. There will be key secondary endpoints measured at 12 weeks, and weight loss will also be measured at 24 and 48 weeks. Participants will be asked to use the intervention materials for the arm to which they were randomized in their weight loss efforts (ie, Keyto self-monitoring device and app or WW app [21]) throughout the 12-, 24-, and 48-week durations of the primary and secondary phases of this trial. The study has been approved by the corresponding author’s university clinical research ethics board. External peer-reviewed funding was secured from Mitacs, a not-for-profit agency that facilitates academic research collaboration with industry partners.

### Participants and Eligibility

We will recruit 144 adults living in the state of California to participate in the study. Please see Table 2 for a list of eligibility criteria. Because of the coronavirus disease 2019 (COVID-19) pandemic, we have also included COVID-19-specific eligibility criteria in Table 2, which were informed by World Health Organization classifications for symptom severity [22]. Eligibility criteria were established to control for extraneous factors and to maintain a relatively homogeneous sample in this trial. For example, participation was constrained to adults who are overweight or obese and without comorbidities to control for potential differential metabolic impacts of a ketogenic on older adults (who would be more likely to have comorbidities or medications) or those with class 3 obesity [23] who might have more complex conditions.

### Recruitment, Screening, Randomization, Enrollment

Participants will be recruited through Facebook advertisements, poster advertisements placed in public spaces (eg, universities, libraries, coffee shops), and emails to the existing Keyto email list. Informed consent will be obtained digitally via Qualtrics (Qualtrics LLC). Following informed consent, participants will schedule a video conference or phone call with a member of the study team who will answer any questions participants may have, clarify the procedures of the study, and schedule a baseline blood test. After collection of the baseline blood sample, participants will be emailed the online baseline questionnaire. Participants will then be randomized by a researcher logging into a password-protected study website and mailed the study materials for their condition. All 144 participants will be randomized at 1:1 ratio to the Keyto (n=72) or Weight Watchers (n=72) trial arms using a variable permuted block sizes, stratifying for sex (male, female) and age (18-40 years, 41-64 years). The randomization website and schedule will be created and maintained by a third party (Centre for Health Evaluation and Outcomes Science, St. Paul’s Hospital, Vancouver, Canada). Participants will be provided with a US $100 gift card to an online store of their choice upon completion of the 48-week follow-up surveys.

**Table 2:** Trial inclusion and exclusion criteria.

Criteria		
**Inclusion**		
	Aged 18-64 years	
	Living in the state of California	
	BMI 27-43 kg/m^2^	
	Must speak, read, and comprehend English	
	Must have a valid email address and phone number	
	Willingness to a follow an evidence-based very low carbohydrate diet or calorie-restricted diet that have been demonstrated for weight loss	
	Must have a kitchen and be willing to cook	
	Willingness to reduce (net) carbohydrate intake to less than 30 g/day	
	Willingness to restrict intake of added sugar, bread, grain, rice, pasta, sweets, most fruits, pastries, and other carbohydrates	
	Willingness to comply with a strict diet for 12 months	
	Interest in losing weight	
	**(COVID-19^a^ specific criteria) If you have been diagnosed with COVID-19, you may be eligible if:**	
		Do not currently have COVID-19 related symptoms
		Your last symptoms were more than 4 weeks ago
		You experienced uncomplicated or mild COVID-19 symptoms
		You were not hospitalized because of your symptoms
**Exclusion**		
	HIV or immunocompromised	
	Current or past cancer diagnosis	
	Pregnant, breastfeeding, or planned pregnancy in next 12 months	
	Beginning or ending hormonal contraception in next 12 months	
	Current diagnosis of diabetes	
	History of heart attack or stent	
	Currently taking glucose-lowering drugs, statins, or oral steroids	
	History of gastric bypass surgery or any other weight-loss surgery	
	History of anorexia or bulimia	
	History of mental illness	
	Current smoker or smoked cigarettes within past 12 months	
	Currently eating fewer than 50 g carbohydrates per day	
	Currently following ketogenic diet or have strictly adhered to a ketogenic diet for greater than 3 weeks in the past 6 months	
	Lost or gained more than 5% body weight in past 6 months	
	Currently using Weight Watchers or have strictly adhered to the WW app for greater than 3 weeks in the past 6 months	
	**(COVID-19 specific criteria) If you have been diagnosed with COVID-19, you may be ineligible if:**	
		You have had moderate to severe COVID-19 associated disease
		You have been hospitalized by COVID-19

^a^COVID-19: coronavirus 2019 disease.

### Interventions

#### Keyto App and Device

Those randomized to the Keyto arm will be asked to download the Keyto app from an app store. They will receive a unique email username and password provided by the study team to log in. Participants will be guided through connecting their iHealth scale and the Keyto app and given a brief overview of its features, with the remainder of the intervention will be delivered entirely via the app. The app contains resource information about what to eat and what not to eat in order to achieve nutritional ketosis and ample resources. The app also contains a social support function. For the trial, all Keyto participants will be placed into a moderated support group of 4 to 6 participants per group. Keyto’s registered dietician and executive medical director will be added to each group to answer questions about Keyto and starting a ketogenic diet. The identities of each user will be kept anonymous and users will be encouraged to refrain from using their names in this forum. Based on the premium version of the Keyto app, a phone or video conference call with the registered dietician will be available in the second week of the program. The app also includes tips and resources about how to succeed potential side effects or pitfalls. There is access to a short podcast offered 3 times per week discussing various aspects of the program including strategies and pitfalls.

The Keyto app uses its Key Eats nutrition plan as a heart-healthy ketogenic diet that emphasizes fish and plant-based fats, moderate protein and nonstarchy vegetables and is compatible with a vegetarian or mixed-diet lifestyle. The app reinforces users to avoid foods with refined carbohydrates like pasta, bread, pizza, or sweets and replacing those with foods high in healthy fats such as avocados, nuts, fatty fish (such as salmon), and olive oil.

The Key Eats Code is a searchable database of almost all foods that rates each using a red, yellow, or green code to indicate whether to eat it *ad libitum*, cautiously, or not at all. The Key Eats Code database was created based on nutritional information gathered from the United States Department of agriculture food database to categorize foods based on the amount of net carbohydrates. Foods labeled as green indicate participants can eat as much as they want; they are very low in carbohydrates and higher in fats. Foods labeled red should be avoided; these are typically high-carbohydrate and high sugar foods like pasta, potatoes, or candy. A yellow label means *think before you eat*. These foods can be eaten occasionally in moderation. There is a *Heart First* badge to indicate foods that are rich in monounsaturated and omega-3 polyunsaturated fats and low in saturated fats. Participants in this trial will be encouraged to eat foods with this badge. There is additionally a *Power Food* badge indicating a food that is especially high in healthy fats and promotes ketosis. For the trial, participants are told to aim to prioritize foods that fall in the green and heart-first category. While conventional ketogenic diets prioritize increased fat intake regardless of source, Keyto emphasizes plant and fish-based fats that are generally lower in saturated fatty acids and higher in unsaturated fatty acids. Research has shown that substituting saturated fats with unsaturated fats can improve cardiovascular disease risk markers [24,25].

Participants will be asked to measure their breath acetone concentrations with the Keyto sensor 3 times daily (immediately after waking up in the morning, before lunch, and before dinner). For this, the participants will be asked to exhale into the hand-held device, which is paired with the participant’s phone. Participants will be able to drink alcohol in moderation but will be instructed to avoid blowing into the Keyto sensor after having an alcoholic beverage.

#### Weight Watchers’ WW app

Those randomized to the Weight Watchers group will be given a unique link to download an app that includes information, resources, and food diary functions for the Weight Watchers diet. They will also receive a unique email and password, provided by the study team, to log in. The research team will pay for the WW app subscription, so there is no cost for participants randomized to the Weight Watchers group. Participants will be guided through connecting the iHealth scale and the WW app and given a brief overview of its features, with the remainder of the intervention will be delivered entirely via the app. Users will get information as to what to eat and what not to eat according to the Weight Watchers diet and points system [21].

The WW app takes a science-backed approach to weight loss that does not involve counting calories or macronutrients. Rather, all foods are assigned a point value. Certain foods are assigned a low number (eg, foods low in calories and fat, like lentils) and others are assigned a high number (eg, foods high in calories and fat, such as pizza). At the start of the program, participants will enter their current and goal weight for the next 12 weeks and complete a baseline assessment to determine current eating habits. Based on this information, participants will be assigned to one of 3 groups within the WW app (green, blue, or purple). Group assignment will determine the number of points allotted for the participant to eat in a day, as well as the number of weekly flex points that can be used if a participant goes over their allotted daily points. The app also contains approximately 100 to 300 zero-point foods (depending on the Weight Watchers group). These are low-calorie foods that participants can eat as much of and do not take any points away from daily or weekly allotted points. Participants record their dietary intake through the app, and the app automatically tracks their point intake and provides feedback to help users stay within their allotted point limit.

The WW app has similar support features as those of Keyto, such as live 24/7 dietary coaching and weight loss groups that participants can join for social support. Additionally, both apps offer recipes and meal plans to improve user experience and aid in dietary adherence. Both apps offer a food search option to allow users to determine if any food fits well into their eating plan.

### Measures

Participants will be asked to respond to survey measures at the 4 outcome time points: baseline and at 12, 24, and 48 weeks from start of the program. Daily and weekly questionnaires and app data will be used as manipulation checks of adherence and intervention fidelity, which are described below.

#### Outcome Measures

##### Body Mass

Participants will be asked to weigh themselves each morning using the Bluetooth weight scale (iHealth Lina H2). Weight measurements will be automatically uploaded to the iHealth cloud that can be accessed by the research team. Body mass measured at baseline, the initial measurement made at the start of the study, and follow-up time points (12, 24, and 48 weeks) will be calculated as the mean of measures taken across the week. A measure of daily adherence to self-weighing will also be examined as the total proportion of days that a participant records their weight.

##### Venous Blood Sample

Participants will be sent to a third-party blood clinic (Quest Diagnostics), to provide a ≥12 hour fasting blood sample obtained by venipuncture from an antecubital vein in the seated position. Blood samples will be obtained at baseline and 12 weeks, with an optional blood sample obtained at 48 weeks. See Table 1 for a complete of list of blood measures including hemoglobin A_1_c, blood glucose, insulin sensitivity, and fasting lipids.

##### 24-Hour Dietary Recall

Participants will complete the Automated Self-Administered 24-Hour Dietary Recall [26] to determine caloric intake and macronutrient composition. Participants will be asked to report all caloric intake within the past 24 hours at baseline and at 12, 24, and 48 weeks. This retrospective questionnaire allows for extraction of detailed information regarding specific nutrients and food groups (eg, carbohydrate intake, consumption of saturated fat). The Automated Self-Administered 24-Hour Dietary Recall has demonstrated high consistency with interviewer assessed 24-hour diet (*r*=0.80) [27] and with 4-day food records [28].

##### Sleep Quality

The Pittsburgh Sleep Quality Index [29] is a validated measure used to assess sleep schedules and sleep quality. The questionnaire assesses quality of sleep from the previous month by measuring the following components: subjective sleep quality, sleep latency, sleep duration, habitual sleep efficiency, sleep disturbances, use of sleep medication, and daytime dysfunction. An overall score will be calculated by totaling these component scores whereby lower scores denote a higher sleep quality. Sleep quality will be assessed at baseline and at 12, 24, and 48 weeks.

#### Demographics

Participant characteristics, including sex, race or ethnicity, education level, occupation, income, marital status, and number of dependents will be collected at baseline via online survey.

#### Manipulation Checks

##### App Data

We will extract app usage data, food logs, breath acetone levels (for the Keyto group only), and weight.

##### Food Attitudes Survey

Participants will complete the Food Attitudes Survey [30], a short, 9-item survey to assess their thoughts and feelings toward food. Participants will be asked, “On a 5-point scale, answer how these comments reflect your thoughts and feelings toward food,” where scores range from 1 (not at all like me) to 5 (exactly like me). An example stem item reads, “It is difficult for me to leave food on my plate.” Attitudes will be measured at baseline and at 12, 24, and 48 weeks.

##### Cravings, Mood, And Energy

This 12-item questionnaire asks participants about the impact of their dietary intervention on aspects of their cravings, mood, and energy in order to determine possible barriers to diet adherence. Participants will be asked, “How does the following impact your ability to stick with your diet?” An example craving response includes, “Having delicious foods in front of you and you can’t resist.” An example mood response includes, “Having a really hard or stressful day.” An example of an energy response includes, “You become too hungry and can’t continue to resist eating.” Responses are rated on a 4-point scale ranging from 1 (not at all) to 4 (every day). As these are manipulation checks and to not overburden participants, 3 of the 12 items will be randomly asked per week. By the primary endpoint at 12 weeks, each participant will have answered each of the 12 questions 3 times. This measure was developed for this study in consultation with a health psychologist and registered dietician.

##### Diet Adherence

Diet adherence will be assessed using 2 questions relating to adherence with the diet participants were randomized to. Participants will be asked, “To what extent do you believe you were able to stick to the diet as part of this study in the past week?” on a 5-point scale ranging from 0 (not at all) to 4 (completely). There will be an open-ended response option for participants to provide an explanation if they wish. Second, participants will be asked, “How often did you monitor and track your food intake on average each day in the past week?” on a 4-point scale ranging from 0 times per day to 3 or more times per day. Participants will be asked about their diet adherence at the end of every week throughout the 48-week trial period.

##### Physical Activity

The Godin Leisure-Time Exercise Questionnaire [31] is a validated and commonly used self-report measure of physical activity. Participants will be asked to report the number of 30-minute bouts of light, moderate, and vigorous physical activity they engaged in over the past week. The Godin Leisure-Time Exercise Questionnaire will be assessed at 12 and 48 weeks.

### Sample Size Calculation

There are no known studies with protocol design as we describe. Thus, the amount of weight loss expected with a hands-off self-monitoring ketogenic mHealth application is not known. Thus, we determined sample size in order to detect a clinically meaningful 5% difference in weight loss, assuming a mean body mass of 100 kg with a standard deviation of 15 kg [32]. A 5% weight loss corresponds to a small-to-moderate effect size (Cohen *d*=0.33, *f*=0.165). Using G*Power software (version 3.1.9.3) a total sample size of 124 is required to detect a between groups effect with 80% power and α=.05 with 2 groups and 2 time points (primary outcome at 12 weeks) assuming a correlation among repeated measures of *r*=0.75. In order to preserve power and account for 15% loss to follow-up, we will aim to recruit 144 participants (ie, n=72 per group).

### Planned Analysis

A statistician independent of the research team will analyze the data. Data will be analyzed on an intention-to-treat basis. Descriptive statistics (mean, SD, and frequency) will be calculated. Univariate and multivariate statistical assumptions will be examined and managed according to recommendations by Tabachnick and Fidell [33]. Mixed linear effects models will be used to assess between-group differences across time. All primary and secondary study outcomes will be analyzed similarly. Follow-up mean comparisons and Cohen *d* effect sizes will used to examine differences between individual time study points. Two rounds of analyses will occur: the primary outcome (body mass) and key secondary outcomes (blood variables, surveys, app data) will be assessed at the 12 weeks and follow-up exploratory analyses will be performed after 24 and 48 weeks. An additional exploratory mixed effects model using all daily body mass measures during the first 12 weeks will examine whether the patterns of body mass change differ between the 2 study groups.

### Potential Risks and Mitigation

The potential risks of this research are minimal and relate to following a low-carbohydrate diet. As with many dietary changes, the switch to a low-carbohydrate diet can sometimes lead to headaches, nausea, or low energy. The Keyto program uses a modified ketogenic or low-carbohydrate high-fat diet which derives 70% of calories from fat. At the beginning of low-carbohydrate high-fat diets, some participants may experience transient adverse side effects including headache, light-headedness, achiness, and muscle cramps [34]. These side effects are sometimes referred to as the “keto flu [35],” appear to be related to changes in electrolytes and diuresis and can be mitigated by broth or bouillon supplementation and adequate sodium intake. The intensity of these symptoms is expected to decline as the study progresses and participants become adapted to a ketogenic diet. Possible long-term adverse side effects of ketogenic diets in adults who are overweight and obese, but otherwise healthy, are currently unknown.

## Results

The study was registered at ClinicalTrials.gov (NCT04165707). Recruitment opened December 1, 2019. The first participants began the app-based diet plan to which they were allocated on January 6, 2020. As of April 2020, there were 49 participants enrolled in the study. We expect rolling recruitment to be completed by July 2020, and the primary intervention phase to be completed 12 weeks later (October 2020), with 2 exploratory follow-up time points at 24 weeks (January 2021) and 48 weeks (July 2021).

## Discussion

Any diet is only as effective as adherence to it. Low-carbohydrate diets can be complex and challenging to adhere to [9,36], and very low–carbohydrate ketogenic diets may be even more challenging as they require greater dietary restriction to promote ketosis and sustain weight loss. Low-carbohydrate and ketogenic diets have been shown to be efficacious at promoting weight loss in structured settings [9,11,37], but maintaining them in free-living conditions may be challenging. This paper outlines the protocol for a randomized trial testing the efficacy of the Keyto breath sensor and app intervention against the WW app in promoting weight loss for 12 weeks. The Keyto intervention is delivered entirely through an app and lowers the burden of self-monitoring required to promote weight loss when following a ketogenic diet by providing an easy means of tracking ketosis using a handheld breath acetone monitor. For this reason, Keyto may be particularly effective at promoting weight loss in free-living conditions.

One potential challenge that may be encountered during the conduct of this trial is strong preferences for one of the diet conditions. Participants with strong diet preferences that are not randomized to their preferred diet may be less motivated to adhere to their allocated condition, which may influence outcomes. In real-world settings, individuals would simply choose the diet plan that they want. However, the design of a randomized trial precludes this possibility. To alleviate this potential confounder, we established eligibility criteria requiring participants to be open to both types of diets. This may constrain the generalizability of our findings to those who are most motivated. However, it is viewed as less of a confounding factor given that both interventions are mHealth products that must be purchased, so users in real-world settings would need to be motivated in order to consider either weight loss app. Furthermore, a randomized efficacy trial with strict eligibility criteria is necessary and appropriate for the stage of research [38]. The findings are generalizable to those meeting the eligibility criteria (eg, motivated adults with overweight or obesity and without comorbidity) and may not be generalizable to the entire population.

This trial will provide evidence comparing weight loss outcomes between a handheld breath ketone monitor and ketogenic diet app (Keyto) and an established caloric restriction–based weight loss app or program (Weight Watchers). The Keyto intervention may help with adherence to a ketogenic diet through direct biofeedback and personalization thereby helping to promote maintained weight loss equivalent or superior to the gold-standard weight loss app. This would provide evidence for breath ketone monitoring as an adjunct to ketogenic diets for adults with obesity who are trying to lose weight.

Research examining changes in metabolic health outcomes following ketogenic diet have typically found a differential impact on blood lipids, with potentially favorable increases in high-density lipoprotein concentrations and decreases in triglycerides observed concomitantly with a potentially unfavorable increase in low-density lipoprotein levels [19]. The heart-healthy approach used by Keyto is novel and is designed to increase consumption of plant- and fish-based foods that are higher in monounsaturated and omega-3 polyunsaturated fatty acids in order protect against an increase in low-density lipoprotein cholesterol. Exploratory blood measures will help to determine whether the heart-healthy ketogenic dietary approach results in changes in blood lipid profile (including low-density lipoprotein particle size distribution) that are favorable for cardiovascular health.

It is important to examine different strategies to combat obesity given its prevalence and adverse impacts. A ketogenic diet may be an effective strategy for weight loss; however, its success depends on adherence, and adherence to a ketogenic diet can be challenging. Furthermore, there are concerns about the potential detrimental cardiovascular effects of the high-fat intake that is promoted in ketogenic diets. This trial will provide high-quality evidence regarding whether Keyto’s version of the ketogenic diet promotes weight loss without detrimental effects on the blood lipid profile.

## Abbreviations

mHealth: mobile health
